# Microwave Assisted Synthesis, Antifungal Activity, and DFT Study of Some Novel Triazolinone Derivatives

**DOI:** 10.1155/2015/916059

**Published:** 2015-03-12

**Authors:** Na-Bo Sun, Jian-Zhong Jin, Fang-Yue He

**Affiliations:** College of Biology and Environmental Engineering, Zhejiang Shuren University, Hangzhou, Zhejiang 310015, China

## Abstract

A series of some novel 1,2,4-triazol-5(4*H*)-one derivatives were designed and synthesized under microwave irradiation via multistep reaction. The structures of 1,2,4-triazoles were confirmed by ^1^H NMR, MS, FTIR, and elemental analysis. The antifungal activities of 1,2,4-triazoles were determined. The antifungal activity results indicated that the compounds **5c**, **5f**, and **5h** exhibited good activity against *Pythium ultimum*, and the compounds **5b** and **5c** displayed good activity against *Corynespora cassiicola*. Theoretical calculation of the compound **5c** was carried out with B3LYP/6-31G (d). The full geometry optimization was carried out using 6-31G(d) basis set, and the frontier orbital energy and electrostatic potential were discussed, and the structure-activity relationship was also studied.

## 1. Introduction

Nowadays, nitrogen-containing heterocycles became a research hot spot because they displayed excellent activities [1–9]. 1,2,4-Triazole derivatives, especially triazolinone compounds, exhibited diverse activities, such as the commercial antidepressant medicine Trazodone, herbicide azafenidin, and herbicides amicarbazone, sulfentrazone, and carfentrazone-ethyl. So the synthesis of substituted triazolinone compounds is one of the important fields for many researchers. Many references reported that triazolinone derivatives showed other interesting activities, including angiotensin II AT(1) receptor antagonists [10–12], anti-human immunodeficiency virus (HIV) activity [13, 14], acetolactate synthase (ALS) inhibitors [15, 16], protoporphyrinogen oxidase inhibitors [17], antioxidant activities [18–20], anticancer activity [21, 22], and anti-inflammatory activity [23].

Microwave-assisted technique is a green method in current organic synthesis [24–28]. It is attractive, offering reduced pollution, low cost, and high yields. The green technique can often shorten the reaction time.

In our previous work [29–33], some 1,2,4-triazole compounds were designed and synthesized. They showed good antifungal activities. In this paper, fifteen novel 1,2,4-triazole derivatives were designed and synthesized under microwave irradiation. Their chemical structures were confirmed by ^1^H NMR, FTIR, MS, and elemental analysis. The antifungal activity of 1,2,4-triazoles was determined* in vivo*.

## 2. Results and Discussion

### 2.1. Synthesis

**Scheme 1:**
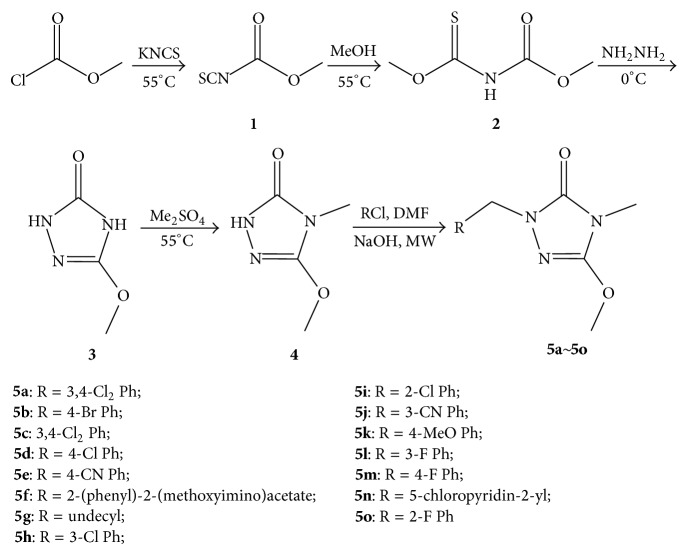
The synthetic route of title compounds.

The synthetic route of target compounds was outlined in Scheme 1. All the reported syntheses of O-methyl carbonisothiocyanatidate involve reaction of a thiocyanate salt (e.g., Pb^2+^, NH_4_^+^, K^+^, and Na^+^) with methyl carbonochloridate. If we use equal molar amounts of potassium thiocyanate and methyl carbonochloridate, approximately equal amounts of both isomers will be obtained. When the methyl carbonochloridate was reacted with potassium thiocyanate, the potassium thiocyanate was excess, while the drop speed must be slow, as fast speed decreased the yield of product. The intermediate** 2** was easily prepared by the reaction of methoxycarbonyl isothiocyanate and methanol. In the synthesis process of intermediate** 3**, the intermediate** 2** cyclized with hydrazine hydrate. Because it is an equilibrium reaction it is reacted under nitrogen atmosphere in order to off hydrogen sulfide gas and increase the yield of intermediate** 3**. The intermediate** 3** exhibits two NH groups, which may be both methylated with (CH_3_)_2_SO_4_. We found that the pH values of two NH groups are different. Therefore, pH value is controlled preferably about 8 to 9 which is given the intermediate** 4**. The target compounds** 5a**~**5o** were synthesized using microwave irradiation method. The signal of NCH_2_ proton appeared around *δ* 3.66–5.04 ppm. The infrared spectrum of the title compounds** 5a**~**5o** showed absorption bands around 2930 cm^−1^ for CH_2_ stretching. The characteristic stretching vibration *ν* (C=O) appears at 1720 cm^−1^. The mass spectrum results showed that molecular ion is in accordance with its molecular formula. The elemental analysis results are in accordance with the calculated results.

### 2.2. Antifungal Activity

The* in vivo* antifungal activity results of 1,2,4-triazol-5(4*H*)-ones against* Phytophthora infestans, Botrytis cinerea, Corynespora cassiicola, Rhizoctonia solani,* and* Pythium ultimum* were shown in Table 1; dimethomorph, fludioxonil, chlorothalonil, validamycin, and zhongshengmycin were used as controls. From Table 1, it is shown that compounds** 5c**,** 5f**, and** 5h** exhibited good control efficacy against* Pythium ultimum* at 500 ppm. Compounds** 5a**,** 5g**,** 5i**,** 5m**, and** 5n** showed moderate control efficacy against* Pythium ultimum*. The control zhongshengmycin had no control efficacy against* Pythium ultimum.* For the* Rhizoctonia solani*, most of the title compounds displayed no control efficacy, except compounds** 5f** and** 5h**. Surprisingly, all the compounds can not only inhibit the* Botrytis cinerea*, but also promote the* Botrytis cinerea* growth. Most of 1,2,4-triazol-5(4*H*)-ones displayed weak control efficacy against* Corynespora cassiicola*; only compounds** 5b** and** 5c** showed good antifungal activity (about 70%) against* Corynespora cassiicola*, which is higher than that of control chlorothalonil. Unfortunately, the title compounds exhibited weak activity against* Phytophthora infestans*.

### 2.3. DFT Calculation

Molecular total energy and frontier orbital energy levels are listed in Table 2. Energy gap between HOMO and LUMO was calculated by B3LYP.

**Figure 1:**
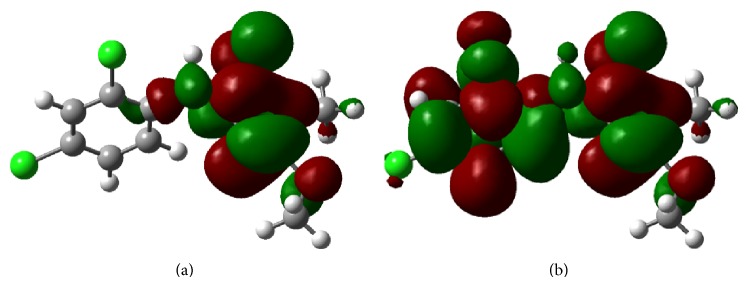
Frontier molecular orbitals of compound** 5c**: (a) LUMO of compound** 5c**; (b) HOMO of compound** 5c**.

According to the frontier molecular orbital theory, HOMO and LUMO are the most important factors that affect the bioactivity. HOMO has the priority to provide electrons, while LUMO can accept electrons firstly [34–36]. Thus, study on the frontier orbital energy can provide useful information about the biological mechanism. From Figure 1, the HOMO of compound** 5c** is mainly located on the OCH_3_ group and 1,2,4-triazol-5(4*H*)-one ring, while the LUMO of compound** 5c** is located on the OCH_3_ group, 2,4-Cl_2_ benzene ring, and 1,2,4-triazol-5(4*H*)-one ring. The fact that the compound** 5c** has strong affinity suggests the importance of the frontier molecular orbital in the *π*-*π* stacking or hydrophobic interactions. From Figure 1, the electron transfer process of the HOMO and LUMO implies that 2,4-Cl_2_ phenyl ring had important impact on the antifungal activity.

**Figure 2:**
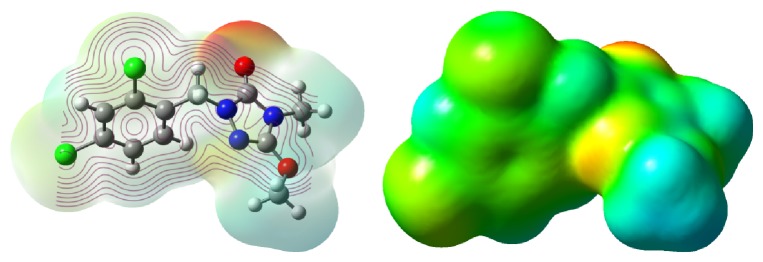
The ESP of compound** 5c**.

The electrostatic potential of compound** 5c** was also calculated. From Figure 2, it is clear that the oxygen atom at the 1,2,4-triazole ring possessed the greatest negative charges and it is therefore possible that the oxygen atom had some interaction with the receptor or acceptor.

Furthermore, the combination of MO provided meaningful clues as to the structural features of these new family fungicides that will be helpful in the design of more potent compounds in the future.

## 3. Materials and Methods

### 3.1. Instruments

Melting points were measured using an X-4 melting apparatus and were uncorrected. ^1^H NMR spectra were determined on a Bruker AC-P400 instrument (400 MHz) using TMS as an internal standard and CDCl_3_ as solvent. Mass spectra were determined on a Thermo Finnigan LCQ Advantage LC/mass detector instrument. Elemental analyses were recorded on a Yanaco MT-3CHN elemental analyzer. Microwave activation was carried out with CEM Discover Focused Microwave (2450 MHz, 300 W). All the reagents are of analytical grade or freshly prepared before use. The course of the reactions was monitored by TLC; analytical TLC was performed on silica gel GF 254.

### 3.2. Synthetic Procedures

The synthetic route is shown in Scheme 1.

#### 3.2.1. Synthesis of Intermediates** 1** and** 2**

The potassium thiocyanate (10.69 g, 0.11 mol) and pyridine (0.40 g) were dissolved in methyl isobutyl ketone (50 mL); methyl chloroformate (9.45 g, 0.10 mol) was added dropwise at 55°C, and the mixture was stirred for 4 h. Then MeOH (20 mL) was added to the mixture and stirred for 16 h. The mixture was washed with concentrated hydrochloric acid (3 mL) and H_2_O (50 mL). After filtration and evaporation of the solvent, the crude intermediate** 2** was collected without further purification: white solid, yield 80%, ^1^H NMR (400 MHz, CDCl_3_) *δ*: 3.77 (s, 3H, COOCH_3_), 4.12 (s, 3H, CSOCH_3_), 8.56 (s, 1H, NH).

#### 3.2.2. Synthesis of Intermediate** 3**

To a solution of intermediate** 2** (50 mmol) in MeOH (75 mL) were added 80% NH_2_NH_2_·H_2_O (4.07 g, 65 mmol) and KOH (45%, 0.81 g, 6.5 mmol) at 0°C; then the mixture was stirred at 30°C for 5 h. After evaporation of the solvent, the crude intermediate** 3** was recrystallized by EtOH to give white crystal** 3**: yield 78%, m.p. 172~173°C, ^1^H NMR (400 MHz, DMSO-*d*_6_) *δ*: 4.02 (s, 3H, triazolone-OCH_3_), 10.51 (s, 1H, NH).

#### 3.2.3. Synthesis of Intermediate** 4**

To a solution of 3 (65 mmol) and K_2_CO_3_ (9.52 g) in CH_3_CN (100 mL) was added (CH_3_)_2_SO_4_ (68 mmol) at 55°C, and the mixture was stirred for another 2 h. The organic phase was extracted with CH_2_Cl_2_ (3 × 10 mL). After drying over sodium sulphate and evaporation of the solvent, the crude was collected without being purified to give the corresponding intermediate** 4**.

#### 3.2.4. General Procedure for Thioether** 5**

DMF (5 mL),** 4** (0.25 g, 1.00 mmol), RCH_2_Cl (1.10 mmol), and NaOH (0.05 g, 1.20 mmol) were charged into a CEM designed 10 mL pressure-rated vial. Then it was irradiated in a CEM Discover Focused Synthesizer (150 w, 90°C, 200 psi, 15 minutes). The mixture was cooled below 50°C. The mixture was poured into crushed ice and the title compound 1,2,4-triazole was collected after being recrystallized.

*1-(3,4-Dichlorobenzyl)-3-methoxy-4-methyl-1H-1,2,4-triazol-5(4H)-one (* ***5a***). m.p. 136–140°C, Yield 88%, ^1^H NMR (400 M, CDCl_3_): 3.16 (s, 3H, N-CH_3_), 3.96 (s, 3H, OCH_3_), 4.83 (s, 2H, NCH_2_), 7.18–7.21 (m, 1H, Ph), 7.40–7.45 (m, 2H, Ph); IR/cm^−1^: 3449.33, 2957.94, 1710.43, 1615.33, 1518.36, 1470.76, 1425.27, 1403.40, 1307.42, 1233.37 1135.45, 1009.21, 914.98, 812.11, 743.63, 662.08, 595.11; ESI-MS: 289 [M+H]^+^. Elemental anal. (%), calculated: C, 45.85; H, 3.85; N, 14.58; found: C, 45.98; H, 3.77; N, 14.43. 

*1-(4-Bromobenzyl)-3-methoxy-4-methyl-1H-1,2,4-triazol-5(4H)-one (* ***5b***). m.p. 138–140°C, Yield 84%, ^1^H NMR (400 M, CDCl_3_): 3.15 (s, 3H, N-CH_3_), 3.95 (s, 3H, OCH_3_), 4.84 (s, 2H, NCH_2_), 7.24 (d,* J* = 6.4 Hz, 2H, Ph), 7.47 (d,* J* = 6.4 Hz, 2H, Ph); IR/cm^−1^: 3450.50, 2942.58, 1711.13, 1607.26, 145.83, 1424.33, 1382.18, 1301.13, 1067.47, 1008.00, 910.76, 847.46, 798.29, 727.42, 600.46; ESI-MS: 299 [M+H]^+^. Elemental anal. (%), calculated: C, 44.31; H, 4.06; N, 14.09; found: C, 44.25; H, 3.92; N, 14.21. 

*1-(2,4-Dichlorobenzyl)-3-methoxy-4-methyl-1H-1,2,4-triazol-5(4H)-one (* ***5c***). m.p. 113–115°C, Yield 89%, ^1^H NMR (400 M, CDCl_3_): 3.17 (s, 3H, N-CH_3_), 3.97 (s, 3H, OCH_3_), 4.99 (s, 2H, NCH_2_), 7.24 (d,* J* = 8.4 Hz, 1H, Ph), 7.22 (d,* J* = 8.4 Hz, 1H, Ph), 7.41 (s, 1H, Ph); IR/cm^−1^: 3440.43, 2943.89, 1710.82, 1606.45, 1522.24, 1490.26, 1455.07, 1424.03, 1382.14, 1318.96, 1229.32, 1091.39, 1006.22, 849.44, 803.25, 599.99; ESI-MS: 289 [M+H]^+^. Elemental anal. (%), calculated: C, 45.85; H, 3.85; N, 14.58; found: C, 45.67; H, 3.77; N, 14.64. 

*1-(4-Chlorobenzyl)-3-methoxy-4-methyl-1H-1,2,4-triazol-5(4H)-one (* ***5d***). m.p. 125–127°C, Yield 82%, ^1^H NMR (400 M, CDCl_3_): 3.15 (s, 3H, N-CH_3_), 3.95 (s, 3H, OCH_3_), 4.80 (s, 2H, NCH_2_), 7.28–7.31 (m, 4H, Ph); IR/cm^−1^: 3430.91, 2954.61, 1723.39, 1611.41, 1517.41, 1470.16, 1414.48, 1352.86, 1302.39, 1230.3, 1050.80, 1010.51, 912.22, 878.42, 829.12, 788.23, 741.01, 691.10, 592.31, 468.63; ESI-MS: 254 [M+H]^+^. Elemental anal. (%), calculated: C, 52.08; H, 4.77; N, 16.56; found: C, 51.89; H, 4.87; N, 16.65. 

*4-((3-Methoxy-4-methyl-5-oxo-4,5-dihydro-1H-1,2,4-triazol-1-yl)methyl)benzonitrile (* ***5e***). m.p. 160–162°C, Yield 89%, ^1^H NMR (400 M, CDCl_3_): 3.15 (s, 3H, N-CH_3_), 3.96 (s, 3H, OCH_3_), 4.94 (s, 2H, NCH_2_), 7.44 (d,* J* = 8.16 Hz, 2H, Ph), 7.64 (d,* J* = 8.16 Hz, 2H, Ph); IR/cm^−1^: 3434.79, 2955.79, 2229.46, 1715.92, 1613.56, 1523.56, 1421.90, 1230.01, 1016.35, 857.47, 735.86, 640.39, 596.36, 552.70; ESI-MS: 245 [M+H]^+^. Elemental anal. (%), calculated: C, 59.01; H, 4.95; N, 22.94; found: C, 58.88; H, 5.12; N, 23.13. 

*(E)-Methyl 2-(2-((3-Methoxy-4-methyl-5-oxo-4,5-dihydro-1H-1,2,4-triazol-1-yl)methyl)phenyl)-2-(methoxyimino)acetate (* ***5f***). m.p. 103–107°C, Yield 89%,^1^H NMR (400 M, CDCl_3_): 3.15 (s, 3H, N-CH_3_), 3.96 (s, 3H, OCH_3_), 4.94 (s, 2H, NCH_2_), 7.44 (d,* J* = 8.16 Hz, 2H, Ph), 7.64 (d,* J* = 8.16 Hz, 2H, Ph); IR/cm^−1^: 3455.02, 2936.45, 1720.46, 1611.72, 1522.17, 1431.39, 1230.32, 1005.55, 86.57, 741.29, 711.75, 681.57, 598.40, 572.80; ESI-MS: 245 [M+H]^+^. Elemental anal. (%), calculated: C, 53.89; H, 5.43; N, 16.76; found: C, 53.98; H, 5.13; N, 16.88. 

*3-Methoxy-4-methyl-1-undecyl-1H-1,2,4-triazol-5(4H)-one (* ***5g***). m.p. 102–105°C, Yield 78%, ^1^H NMR (400 M, CDCl_3_): 0.86 (t,* J* = 6.71 Hz, 3H, CH_3_), 1.24–1.29 (m, 16H, CH_2_), 1.66–1.68 (m, 2H, CH_2_), 3.10 (s, 3H, N-CH_3_), 3.66 (s,* J* = 7.21 Hz, 2H, NCH_2_), 3.95 (s, 3H, OCH_3_); IR/cm^−1^: 3446.77, 2941.81, 1708.02, 1622.82, 1258.48, 1131.46, 1101.03, 763.78, 661.94; ESI-MS: 285 [M+H]^+^. Elemental anal. (%), calculated: C, 63.57; H, 10.31; N, 14.83; found: C, 63.76; H, 10.52; N, 14.97. 

*1-(3-Chlorobenzyl)-3-methoxy-4-methyl-1H-1,2,4-triazol-5(4H)-one (* ***5h***). m.p. 97–100°C, Yield 90%, ^1^H NMR (400 M, CDCl_3_): 3.17 (s, 3H, N-CH_3_), 3.96 (s, 3H, OCH_3_), 4.87 (s, 2H, NCH_2_), 7.27–7.34 (m, 4H, Ph); IR/cm^−1^: 3441.67, 2960.00, 1716.14, 1621.83, 1520.80, 1391.88, 1267.10, 1228.92, 014.96, 789.51, 740.97, 593.84; ESI-MS: 254 [M+H]^+^. Elemental anal. (%), calculated: C, 52.08; H, 4.77; N, 16.56; found: C, 52.21; H, 4.87; N, 16.77. 

*1-(2-Chlorobenzyl)-3-methoxy-4-methyl-1H-1,2,4-triazol-5(4H)-one (* ***5i***). m.p. 120–122°C, Yield 87%, ^1^H NMR (400 M, CDCl_3_): 3.19 (s, 3H, N-CH_3_), 3.97 (s, 3H, OCH_3_), 5.04 (s, 2H, NCH_2_), 7.19–7.25 (m, 2H, Ph), 7.37–7.39 (m, 2H, Ph); IR/cm^−1^: 3439.30, 2947.24, 1718.79, 1614.24, 1523.69, 745.45, 595.23; ESI-MS: 254 [M+H]^+^. Elemental anal. (%), calculated: C, 52.08; H, 4.77; N, 16.56; found: C, 52.22; H, 4.88; N,6.67. 

*3-((3-Methoxy-4-methyl-5-oxo-4,5-dihydro-1H-1,2,4-triazol-1-yl)methyl)benzonitrile (* ***5j***). m.p. 130°C, Yield 89%, ^1^H NMR (400 M, CDCl_3_): 3.17 (s, 3H, N-CH_3_), 3.97 (s, 3H, OCH_3_), 4.91 (s, 2H, NCH_2_), 7.46–7.48 (m, 1H, Ph), 7.58–7.62 (m, 3H, Ph); IR/cm^−1^: 3446.70, 2946.55, 2231.74, 1706.17, 1522.05, 1383.55, 1229.24, 1007.72, 786.75, 743.50, 702.92; ESI-MS: 245 [M+H]^+^. Elemental anal. (%), calculated: C, 59.01; H, 4.95; N, 22.94; found: C, 58.88; H, 4.89; N, 22.78. 

*3-Methoxy-1-(4-methoxybenzyl)-4-methyl-1H-1,2,4-triazol-5(4H)-one (* ***5k***). m.p. 88–90°C, Yield 93%, ^1^H NMR (400 M, CDCl_3_): 3.14 (s, 3H, N-CH_3_), 3.81 (s, 3H, OCH_3_), 3.95 (s, 3H, OCH_3_), 4.82 (s, 2H, NCH_2_), 6.87 (d,* J* = 8.14 Hz, 2H, Ph), 7.31 (d,* J* = 8.14 Hz, 2H, Ph); IR/cm^−1^: 3444.95, 2924.39, 1721.08, 1612.50, 1523.71, 1434.36, 1230.54, 742.97, 598.46; ESI-MS: 250 [M+H]^+^. Elemental anal. (%), calculated: C, 57.82; H, 6.07; N, 16.86; found: C, 58.01; H, 6.21; N, 16.76. 

*1-(3-Fluorobenzyl)-3-methoxy-4-methyl-1H-1,2,4-triazol-5(4H)-one (* ***5l***). m.p. 102-103°C, Yield 87%, ^1^H NMR (400 M, CDCl_3_): 3.14 (s, 3H, N-CH_3_), 3.94 (s, 3H, OCH_3_), 4.86 (s, 2H, NCH_2_), 6.96–7.30 (m, 4H, Ph); IR/cm^−1^: 3448.98, 2936.21, 1720.91, 1612.49, 1517.26, 1244.10, 1018.13, 595.24; ESI-MS: 238 [M+H]^+^. Elemental anal. (%), calculated: C, 55.69; H, 5.10; N, 17.71; found: C, 55.79; H, 5.12; N, 17.77. 

*1-(4-Fluorobenzyl)-3-methoxy-4-methyl-1H-1,2,4-triazol-5(4H)-one (* ***5m***). m.p. 102-103°C, Yield 91%, ^1^H NMR (400 M, CDCl_3_): 3.15 (s, 3H, N-CH_3_), 3.95 (s, 3H, OCH_3_), 4.97 (s, 2H, NCH_2_), 7.05–7.12 (m, 2H, Ph), 7.27–7.29 (m, 2H, Ph); IR/cm^−1^: 3427.88, 2956.26, 1709.34, 1611.3, 1519.26, 1453.60, 1399.75, 1235.67, 1134.51, 1012.75, 783.65, 745.36, 700.77; ESI-MS: 238 [M+H]^+^. Elemental anal. (%), calculated: C, 55.69; H, 5.10; N, 17.71; found: C, 55.76; H, 5.22; N, 17.89. 

*1-((5-Chloropyridin-2-yl)methyl)-3-methoxy-4-methyl-1H-1,2,4-triazol-5(4H)-one (* ***5n***). m.p. 111–113°C, Yield 88%, ^1^H NMR (400 M, CDCl_3_): 3.15 (s, 3H, N-CH_3_), 3.95 (s, 3H, OCH_3_), 4.48 (s, 2H, NCH_2_), 7.30–7.33 (m, 1H, Py), 7.68(d,* J* = 8.0 Hz, 1H, Py), 8.42 (s, 1H, Py); IR/cm^−1^: 3436.68, 2955.93, 1711.34, 1611.26, 1519.05, 1399.59, 1235.83, 1134.81, 1012.73, 783.64, 701.50; ESI-MS: 256 [M+H]^+^. Elemental anal. (%), calculated: C, 47.16; H, 4.35; N, 22.00; found: C, 47.32; H, 4.44; N, 22.12.

*1-(2-Fluorobenzyl)-3-methoxy-4-methyl-1H-1,2,4-triazol-5(4H)-one (* ***5o***). m.p. 103-104°C, Yield 91%, ^1^H NMR (400 M, CDCl_3_): 3.19 (s, 3H, N-CH_3_), 3.97 (s, 3H, OCH_3_), 5.04 (s, 2H, NCH_2_), 7.19–7.22 (m, 1H, Ph), 7.24–7.28 (m, 2H, Ph), 7.37–7.39 (m, 1H, Ph); IR/cm^−1^: 3436.50, 2934.06, 1716.72, 1613.09, 1521.04, 1416.95, 1299.19, 1230.77, 1106.39, 1004.22, 826.93, 595.53; ESI-MS: 238 [M+H]^+^. Elemental anal. (%), calculated: C, 55.69; H, 5.10; N, 17.71; found: C, 55.47; H, 5.12; N, 17.88.

### 3.3. Antifungal Activities

The biological activities of title compounds against* Phytophthora infestans, Botrytis cinerea, Corynespora cassiicola, Rhizoctonia solani,* and* Pythium ultimum* were evaluated according to [37–40], and a potted plant test method was adopted.

Germination was conducted by soaking cucumber seeds in water for 2 h at 50°C and then keeping the seeds moist for 24 h at 28°C in an incubator. When the radicles were 0.5 cm, the seeds were grown in plastic pots containing a 1 : 1 (v/v) mixture of vermiculite and peat. Cucumber and tomato plants used for inoculations were at the stage of two seed leaves. Tested compounds and commercial fungicides were sprayed with a hand spray on the surface of the seed leaves on a fine morning at the standard concentration of 500 *μ*g/mL; dimethomorph, fludioxonil, chlorothalonil, validamycin, and zhongshengmycin were used as control. After 2 h, inoculation of* Phytophthora infestans* was carried out by spraying fungal suspension, inoculation of* Rhizoctonia solani* and* Corynespora cassiicola* was carried out by spraying mycelial suspension, and inoculation of* Botrytis cinerea* was carried out by radicle soaking.* Pythium ultimum* was found in the cucumber in nature. The experiment was repeated 4 times. After inoculation, the plants were maintained at 18–30°C (mean temperature of 24°C and above 80% relative humidity (RH)). The fungicidal activity was evaluated when the nontreated cucumber plant (blank) fully developed symptoms. The area of inoculated treated leaves covered by disease symptoms was assessed and compared to that of nontreated ones to determine the average disease index. The relative control efficacy of compounds compared to the blank assay was calculated via the following equation:

(1) $$\begin{array}{c} \;\text{relative}\;\text{control}\;\text{efficacy}\;\left(\%\right)=\frac{\left(\text{CK}-\text{PT}\right)}{\text{CK}}\times 100\%, \end{array}$$

where CK is the average disease index during the blank assay and PT is the average disease index after treatment during testing.

### 3.4. DFT Calculation

DFT-B3LYP/6-31G (d) methods in Gaussian 03 package [41] were used to optimize the structure of** 5c**. Vibration analysis showed that the optimized structures were in accordance with the minimum points on the potential energy surfaces. All the convergent precisions were the system default values, and all the calculations were carried out on the DELL personal computer.

## 4. Conclusion

In summary, this paper reported some novel 1,2,4-triazol-5(4*H*)-one derivatives were successfully synthesized. The bioassay results showed that some of the title compounds exhibited considerable antifungal activity. The bioactivity of these novel compounds deserves further investigation.

## Figures and Tables

**Table 1:** The antifungal activity of title compounds *in vivo* at 500 ppm (%).

Number	*Phytophthora infestans *	*Botrytis cinerea *	*Corynespora cassiicola *	*Rhizoctonia solani *	*Pythium ultimum *
**5a**	25.66	−37.95	19.81	0	44.44
**5b**	−0.8	−61.1	68.6	3.33	−11.11
**5c**	−0.8	−61.1	73.93	0	77.78
**5d**	−0.8	−49.52	26.26	0	11.11
**5e**	−0.8	−38.91	13.08	0	−88.89
**5f**	−0.8	−51.45	18.13	33.89	88.89
**5g**	−0.8	−56.28	9.72	2.22	33.33
**5h**	−0.8	−52.42	6.36	29.44	66.67
**5i**	−0.8	−38.91	37.76	0	44.44
**5j**	−0.8	−48.56	39.44	0	−11.11
**5k**	−0.8	−33.12	34.95	0	−22.22
**5l**	29.16	−27.34	37.76	0	−22.22
**5m**	−0.8	−7.08	24.86	0	33.33
**5n**	−0.8	−42.77	0.19	11.11	33.33
**5o**	−0.8	−20.58	4.11	0	11.11
Dimethomorph	97.76				
Fludioxonil		86.98			
Chlorothalonil			45.89		
Validamycin				62.50	
Zhongshengmycin					0

**Table 2:** Total energy and frontier orbital energy.

	DFT
*E*_total_/Hartree^b^	−1660.90340104
*E*_HOMO_/Hartree	−0.21696
*E*_LUMO_/Hartree	−0.02347
Δ*E* ^a^/Hartree	0.19349

^a^Δ*E* = *E*_LUMO_ − *E*_HOMO_.

^b^1 Hartree = 4.35974417 × 10^−18^ J = 27.2113845 ev.
